# 阵发性睡眠性血红蛋白尿症患者血细胞表面补体沉积水平及与临床症状的相关性

**DOI:** 10.3760/cma.j.cn121090-20240717-00265

**Published:** 2025-04

**Authors:** 梦露 张, 玄 汪, 辰 杨, 苗 陈, 冰 韩

**Affiliations:** 中国医学科学院北京协和医院血液内科，北京 100730 Department of Hematology, Peking Union Medical College Hospital, Chinese Academy of Medical Sciences, Beijing 100730, China

**Keywords:** 血红蛋白尿，阵发性, 溶血, 血栓形成, 补体, 流式细胞术, Hemoglobinuria, paroxysmal, Hemolysis, Thrombosis, Complement, Flow cytometry

## Abstract

**目的:**

探索阵发性睡眠性血红蛋白尿症（PNH）患者血细胞表面补体沉积水平及与临床症状的相关性。

**方法:**

回顾性收集2021年2月至2023年2月就诊于北京协和医院未经补体抑制剂治疗的PNH患者的临床资料，留取患者外周血样本。选取性别及年龄匹配的正常人作为对照组（NC）。通过流式细胞术检测患者及NC外周血红细胞、白细胞和血小板表面C5b-9、C3、C4b及B因子（FB）的沉积水平，并分析其与患者临床症状的相关性。

**结果:**

共纳入73例PNH患者，男42例（57.5％），中位年龄36（14～76）岁，NC 16名。PNH患者中，36例（49.3％）为经典型PNH，37例（50.7％）为再生障碍性贫血-PNH综合征；18例（24.7％）曾发生血栓栓塞事件（TEE）。患者中位HGB为76（37～116）g/L，中位网织红细胞绝对计数（Ret）为181.0（45.9～495.8）×10^9^/L，中位乳酸脱氢酶（LDH）为1 875（377～5 509）U/L；中位Flaer^-^白细胞为94.0％（13.0％～99.9％），中位CD59^-^红细胞为46.7％（9.0％～93.0％）。患者红细胞、白细胞和血小板表面C5b-9、C3、C4b和FB的沉积均显著高于NC（*P*值均<0.05）。患者上述三类血细胞表面C5b-9的沉积均明显高于C3、C4b和FB（*P*值均<0.01）；所有补体片段在红细胞表面的沉积均低于白细胞和血小板（*P*值均<0.01）。患者红细胞表面C5b-9的沉积水平与Ret呈正相关（*P*＝0.005）；发生TEE者白细胞及血小板表面的C3沉积、白细胞表面的C4b沉积水平均低于未发生TEE者（*P*值分别为0.001、0.002、0.017）。

**结论:**

PNH患者血细胞表面C5b-9、C3、C4b和FB的沉积均高于NC，红细胞表面C5b-9水平高可提示活跃溶血，白细胞表面的C3、C4b及血小板表面的C3沉积水平低可提示TEE风险。

## Abstract (en)

**Objective:**

To explore the complement deposition levels on blood cell surfaces in patients with paroxysmal nocturnal hemoglobinuria（PNH）and evaluate their association with clinical manifestations.

**Methods:**

This study enrolled patients with PNH, who had not been treated with complement inhibitors and appeared at Peking Union Medical College Hospital from February 2021 to February 2023. The clinical information of participants was retrospectively recorded, and peripheral blood samples were collected. Gender- and age-matched normal controls（NC）were recruited accordingly. C5b-9, C3, C4b, and factor B（FB）deposition levels on peripheral red blood cells, white blood cells, and platelets were detected with flow cytometry. The correlation between complement deposition levels and clinical symptoms was analyzed.

**Results:**

This study involved 73 patients with PNH, including 42（57.5％）males, with a median age of 36（range: 14–76）years. 16 matched NC were collected. Among patients with PNH, 36（49.3％）had classical PNH and 37（50.7％）had aplastic anemia-PNH syndrome. Thromboembolic events（TEE）occurred in 18（24.7％）patients. The median HGB, absolute reticulocyte count（Ret）, and lactate dehydrogenase of PNH patients were 76（37–116）g/L, 181.0（45.9–495.8）×10^9^/L, and 1 875（377–5 509）U/L, respectively. The median number of Flaer-negative white blood cells was 94.0％（13.0％–99.9％）; the median CD59 negative red blood cells was 46.7％（9.0％–93.0％）. The deposition of C5b-9, C3, C4b, and FB on red blood cells, white blood cells, and platelets in patients with PNH was significantly higher than that in NC（all *P*<0.05）. C5b-9 deposition level was significantly higher than that of C3, C4b, and FB on all three blood cell lineages in PNH patients（all *P*<0.01）. The deposition of all complement fragments on red blood cells was significantly lower than that on white blood cells and platelets（all *P*<0.01）. C5b-9 deposition on red blood cells was positively correlated with Ret in PNH patients（*P*＝0.005）. C3（*P*＝0.001）and C4b（*P*＝0.017）deposition levels on white blood cells and C3 deposition on platelets（*P*＝0.002）in patients with TEE history were lower than those without.

**Conclusion:**

C5b-9, C3, C4b, and FB deposition levels on all three blood cells in patients with PNH were higher than NC. Increased C5b-9 on red blood cells may indicate active hemolysis. Reduced C3 and C4b levels on white blood cells and low C3 deposition on platelets may indicate TEE risk.

阵发性睡眠性血红蛋白尿症（PNH）是一种获得性克隆性造血干细胞（HSC）疾病。磷脂酰肌醇聚糖锚生物合成A类（PIG-A）基因突变造成HSC表面CD55及CD59缺陷[1]–[3]。CD59为补体终末通路抑制分子，可抑制膜攻击复合物（MAC）的形成，CD59缺失的红细胞受MAC攻击裂解，造成血管内溶血（IVH）[4]–[5]。CD55可抑制C3转化酶，在更上游的位置抑制补体激活，CD55缺失的红细胞易出现C3蓄积，产生血管外溶血（EVH）[6]。PNH的主要临床表现包括溶血及血栓栓塞事件（TEE），若合并骨髓衰竭（BMF），还将出现贫血、血小板减少等表现[3],[5]。

在IVH患者中，通过酶联免疫吸附测定（ELISA）检测红细胞表面C5b-9的沉积，仅在破碎红细胞上检测出C5b-9，完整红细胞上尚未检测出；且发生溶血前未检测出白细胞表面C5b-9沉积，在发生溶血后可检测到，说明红细胞及白细胞表面结合的C5b-9在一定程度上能提示溶血，尤其是破碎红细胞结合的C5b-9[7]。既往研究通过流式细胞术（FCM）检测患者红细胞表面C5b-9沉积，发现患者完整红细胞表面C5b-9水平较正常对照组（NC）明显升高，且破碎红细胞表面沉积了更多的C5b-9[8]。表明尽管完整红细胞表面结合的C5b-9有限，仍然与正常人存在差异，且与溶血的发生显著相关。但其他血细胞表面的C5b-9、血细胞表面C4和B因子（FB）沉积与PNH患者临床表现的相关性仍不明确，也未见这些补体片段沉积与临床表现相关性的研究。

TEE是PNH患者死亡的首要原因[9]–[10]，其产生机制复杂，包括补体沉积介导的血小板及白细胞激活、IVH、血小板及内皮细胞释放微粒体（MP）等[9]–[10]。体外研究发现，C5b-9介导的PNH红细胞克隆释放的MP有促凝作用，也可能是PHN患者易发生TEE的原因之一[11]。关于血细胞表面补体沉积与TEE相关性的研究罕见。有临床研究显示，患者CD59^-^血小板上C5b-9的沉积水平较患者的正常血小板（CD59^+^血小板）及对照组血小板均明显升高，PNH患者CD59^-^血小板上C5b-9的沉积可能导致血小板激活，使患者易发生TEE[12]–[13]；但由于发生TEE患者例数有限，该研究未能明确TEE与补体沉积的相关性。

本研究中，我们检测了血细胞（红细胞、白细胞和血小板）表面C5b-9、C3、C4b及FB的沉积水平，探究其与患者临床症状的相关性。

## 病例与方法

1. 病例：本研究纳入2021年2月至2023年2月在北京协和医院血液科门诊就诊的PNH患者，PNH的诊断及分型参照2016年美国PNH诊疗共识[14]。本研究仅纳入经典PNH及再生障碍性贫血（AA）-PNH综合征患者。

纳入本研究的患者还需排除以下情况：①入组前接受过补体抑制剂治疗；②已知或疑似遗传性或获得性补体缺乏，目前或曾有活动性原发性或继发性免疫缺陷病史；③脾切除史，骨髓、HSC或实体器官移植史；④肝炎、结核等感染病史；⑤过去5年内有任何器官或系统的恶性肿瘤史；⑥严重合并症，如重度肾病、晚期心脏病、重度肺动脉高压等事件。

同时收集年龄、性别匹配的NC的外周血标本。

本研究已经北京协和医院伦理委员会批准（伦理批号：HS-3036），所有患者均签署知情同意书。

2. 临床资料收集：收集所有入组PNH患者的临床资料，包括年龄、性别、疾病病程、临床表现、合并症、用药史、既往史、体格检查等。完善PNH相关实验室检查，包括血常规、网织红细胞绝对计数（Ret）、肝肾功能、铁蛋白（Fer）、D-二聚体（D-dimer）、PNH克隆（CD59^-^红细胞、CD55^-^红细胞、CD59^-^白细胞及Flaer^-^白细胞）。完善骨髓涂片、骨髓活检、染色体检查。TEE的诊断包括临床表现、实验室检查（凝血功能、血小板聚集、血栓弹力图等）及影像学检查（B超、CT、血管造影等）。

3. FCM分析：采集外周血样本于枸橼酸钠抗凝管中，24 h内用FACSCantoⅡ流式细胞仪（美国BD公司产品）上机检测。通过双抗体染色FCM检测红细胞、白细胞、血小板上C5b-9、C3、C4b及FB的沉积水平。

将试管分别标记1、2、3，每管分别加入等量样本（检测时取红细胞样本2.5 µl、白细胞样本100 µl、适量血小板样本以100×*g*离心5 min后取上清10 µl）。管1加入异硫氰酸荧光素（FITC）标记的抗C3抗体（美国Abcam公司产品）、PE标记的抗C4b抗体（美国Thermo Fisher科技公司产品），管2加入藻红蛋白（PE）标记的抗FB抗体（美国Thermo Fisher科技公司产品），管3按推荐量加入PE标记的抗C5b-9抗体（美国Thermo Fisher科技公司产品）。测红细胞表面补体沉积时，3管均加入磷酸盐缓冲液（PBS），定容至100 µl，涡旋混匀，避光孵育30 min。测白细胞时，3管均加入PerCP-Cy5.5标记的抗CD15抗体（美国Biolegend公司产品），涡旋混匀避光孵育30 min。加入1 ml含固定剂的溶血素，涡旋混匀，避光孵育10 min，1500 r/min离心10 min弃上清，富集细胞。测血小板时，3管均加入PerCP-Cy5.5标记的抗CD61抗体（美国Biolegend公司产品），均加入90 µl PBS，将染色体积定容至100 µl，涡旋混匀避光孵育30 min。加入2 ml PBS溶液，涡旋混匀，1500 r/min离心5 min，弃上清富集细胞。加入200 µl PBS溶液，涡旋混匀后立即上机。

4. 统计学处理：连续型变量以“*M*（范围）”或“*x*±*s*”表示，分类变量以“例数（％）”表示。不同细胞表面及不同补体片段沉积水平的比较采用相关样本Friedman秩和检验，补体沉积水平与临床指标相关性分析采用Pearson相关性检验，连续型变量组间比较采用独立样本*t*检验。分类变量的组间比较采用卡方检验或Fisher精确检验。所有统计学检验均为双侧检验，*P*<0.05为差异有统计学意义。采用SPSS Statistics 26.0进行数据分析及作图。

## 结果

1. PNH患者的临床特征：本研究共纳入73例PNH患者，男42例（57.5％），中位年龄36（14～76）岁。其中36例（49.3％）为经典型PNH，37例（50.7％）为AA-PNH综合征，无亚临床型PNH。自诊断到采集样本的时间为51（4～490）个月。18例（24.3％）有TEE发生史，其中15例（20.5％）发生1次TEE，1例（1.4％）发生2次，2例（2.8％）发生≥3次。既往用药包括：27例（36.8％）应用免疫抑制剂（如环孢素A、他克莫司、吗替麦考酚酯），53例（73.7％）应用糖皮质激素，56例（76.3％）应用雄激素，17例（23.7％）应用造血刺激因子，27例（36.8％）应用铁剂，其他药物还包括艾曲波帕、罗沙司他、低分子肝素、华法林、利伐沙班等。

患者中位HGB为76（37～116）g/L，中位Ret为181.0（45.9～495.8）×10^9^/L，中位WBC为4.0（1.7～19.3）×10⁹/L，中位中性粒细胞计数2.2（0.4～16.6）×10⁹/L，中位PLT为151（11～378）×10⁹/L，中位血肌酐67（16～249）µmol/L，中位ALT为18（7～54）U/L，中位GGT为15（7～80）U/L，中位总胆红素（TBIL）为34.2（8.1～200.2）µmol/L，中位LDH为1 875（377～5 509）U/L，中位Fer为24（6～5 172）µg/L，中位D-dimer为0.51（0.15～4.79）mg/L。PNH白细胞克隆以Flaer^-^白细胞表示，中位比例为94.0％（13.0％～99.9％）；PNH红细胞克隆以CD59^-^红细胞表示，中位比例为46.7％（9.0％～93.0％）。

2. PNH患者及NC血细胞表面补体沉积水平：首先利用侧向散射（SSC）、PerCP-Cy5.5标记的抗CD15抗体及PerCP-Cy5.5标记的抗CD61抗体分别圈出红细胞、白细胞及血小板细胞群，再用PE标记的抗C5b-9抗体、FITC标记的抗C3抗体、PE标记的抗C4b抗体、PE标记的抗FB抗体检测这些补体片段分子在圈定细胞群表面的沉积水平。图1A显示，PNH患者红细胞、白细胞及血小板表面各补体片段均有一定水平的沉积。图1B以1名正常人为例，其红细胞、白细胞及血小板表面各补体分子的沉积水平几乎为0。分析发现，PNH患者红细胞、白细胞及血小板表面C5b-9、C3、C4b和FB的沉积水平均较NC明显升高（*P*<0.05），提示PNH患者整体补体活性较NC升高（表1）。进一步比较PNH患者不同血细胞表面各补体片段沉积水平，发现三类血细胞表面C5b-9的沉积水平均明显高于C3、C4b和FB（*P*值均<0.001），提示在使用补体抑制剂之前，终末通路（C5b-9）补体活性较上游的经典途径（C4b）和旁路途径（C3、FB）更高。此外，红细胞表面C5b-9、C3、C4b和FB的沉积水平均明显低于白细胞和血小板（*P*<0.01）（表2）。

**图1:**
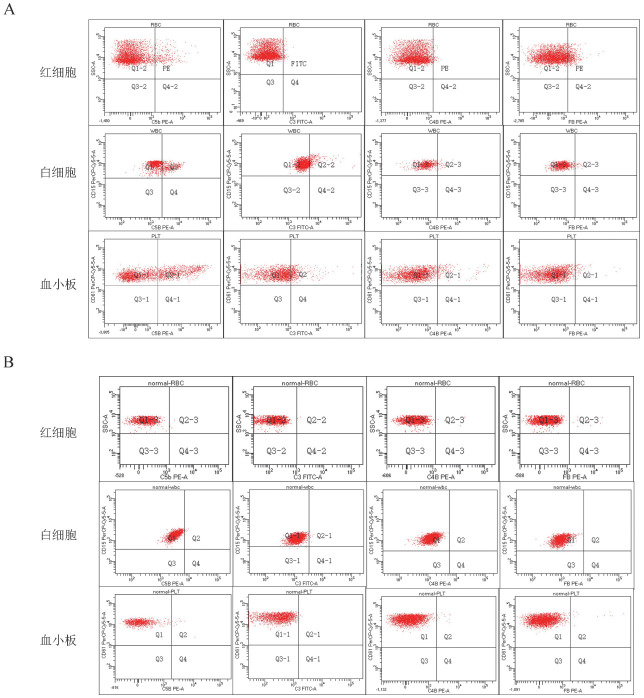
双抗体染色流式细胞术检测1例阵发性睡眠性血红蛋白尿症患者（A）和1名正常对照（B）红细胞、白细胞、血小板表面补体沉积水平

**表1:** 正常对照组与阵发性睡眠性血红蛋白尿症（PNH）患者血细胞表面补体沉积水平比较（％，*x*±*s*）

细胞类型	补体片段	正常对照组（16例）	PNH患者（71例）	*t*值	*P*值
红细胞	C5b-9	0.5±0.3	3.1±2.3	−9.18	<0.001
	C3	0.1±0.0	0.9±1.5	−4.49	0.002
	C4b	0.2±0.2	0.6±0.9	−3.10	0.002
	B因子	0.2±0.2	0.7±0.9	−2.66	0.018
白细胞	C5b-9	0.3±0.2	5.2±3.9	−10.53	<0.001
	C3	0.4±0.4	2.6±3.1	−5.59	<0.001
	C4b	0.1±0.1	2.0±1.9	−9.02	<0.001
	B因子	0.1±0.1	1.2±1.3	−7.36	<0.001
血小板	C5b-9	0.4±0.3	5.0±5.5	−6.78	<0.001
	C3	0.6±0.4	2.7±4.1	−3.91	0.002
	C4b	0.2±0.2	2.1±3.6	−4.07	<0.001
	B因子	0.2±0.2	2.8±4.1	−5.32	<0.001

**表2:** 阵发性睡眠性血红蛋白尿症患者不同血细胞表面各补体片段沉积水平比较（％，*x*±*s*）

补体片段	红细胞（63例）	白细胞（71例）	血小板（71例）	*χ*^2^值^a^	*P*值^a^
C5b-9	3.1±2.3	5.2±3.9	5.0±5.5	30.8	<0.001
C3	0.9±1.5	2.6±3.1	2.7±4.1	31.9	<0.001
C4b	0.6±0.9	2.0±1.9	2.1±3.6	37.2	<0.001
B因子	0.7±0.9	1.2±1.3	2.8±4.1	22.2	<0.001

*χ*^2^值^b^	41.8	107.6	36.7	–	–
*P*值^b^	<0.001	<0.001	<0.001	–	–

注 ^a^不同血细胞表面同一补体片段沉积水平比较；^b^同种血细胞表面不同补体片段沉积水平比较；–：无数据

3. PNH患者血细胞表面补体沉积水平与临床指标的相关性：进一步探索PNH患者红细胞、白细胞及血小板表面各补体沉积水平与临床指标（HGB、Ret、TBIL、LDH、D-dimer、Flaer^-^白细胞、CD59^-^红细胞等）的相关性。结果显示，C5b-9^+^红细胞与Ret呈正相关（*r*＝0.381，*P*＝0.005）（图2）。经典型PNH患者的C5b-9^+^白细胞［（5.5±3.8）％对（3.9±2.6）％，*P*＝0.043］、C3^+^白细胞［（3.4±4.5）％对（1.5±1.3）％，*P*＝0.015］及C3^+^血小板［（3.4±5.9）％对（1.3±1.6）％，*P*＝0.049］水平明显高于AA-PNH综合征患者，提示经典型PNH的补体激活水平可能高于AA-PNH综合征。未发现补体沉积水平与其他临床指标的相关性（*P*值均>0.05）。

**图2:**
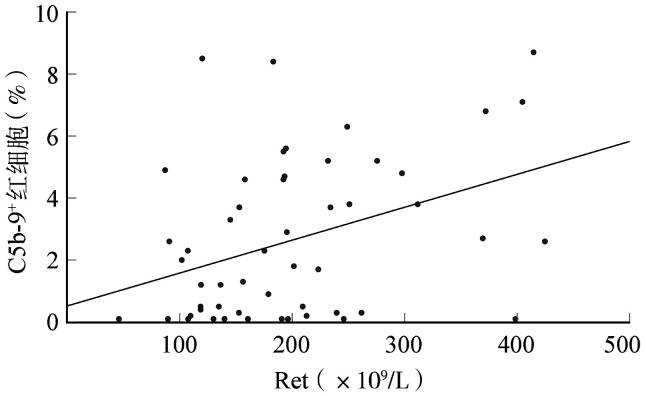
63例阵发性睡眠性血红蛋白尿症患者C5b-9^+^红细胞与网织红细胞绝对计数（Ret）的相关性

4. PNH患者发生TEE与血细胞补体沉积水平的相关性：根据是否曾发生TEE将患者分为无TEE组（55例）及TEE组（18例）。比较两组补体沉积水平，发现与未发生TEE患者相比，发生TEE患者的C3^+^白细胞［（1.0±1.0）％对（2.9±3.8）％，*P*＝0.001］、C4b^+^白细胞［（1.1±1.1）％对（2.1±2.0）％，*P*＝0.017］及C3^+^血小板［（0.6±1.1）％对（2.2±4.0）％，*P*＝0.002］水平更低。两组患者红细胞表面补体沉积水平及三类血细胞表面C5b-9和FB沉积水平的差异均无统计学意义（*P*值均>0.05）（表3）。

**表3:** 无血栓栓塞事件（TEE）组与TEE组患者血细胞表面补体沉积水平比较（％，*x*±*s*）

细胞类型及补体片段	无TEE组（55例）	TEE组（18例）	*t*值	*P*值
FLAER^-^白细胞	82.6±22.5	88.0±22.0	−0.90	0.380
CD59^-^红细胞	47.3±23.5	50.9±25.8	−0.53	0.576
红细胞				
C5b-9	2.1±2.2	3.1±2.9	1.34	0.123
C3	1.1±2.4	0.6±1.1	1.21	0.414
C4b	0.7±0.9	0.4±0.7	1.47	0.215
B因子	0.6±0.8	0.5±0.9	0.42	0.611
白细胞				
C5b-9	4.8±3.5	4.3±2.4	0.68	0.556
C3	2.9±3.8	1.0±1.0	3.37	0.001
C4b	2.1±2.0	1.1±1.1	2.67	0.017
B因子	1.2±1.4	0.7±0.9	1.76	0.158
血小板				
C5b-9	4.6±6.5	3.3±2.9	1.17	0.408
C3	2.9±4.9	0.6±1.1	3.24	0.002
C4b	2.2±4.0	0.7±1.1	2.51	0.121
B因子	2.7±4.6	0.8±1.3	2.75	0.087

## 讨论

2022年，第一代C5补体抑制剂Eculizumab（依库珠单抗）在中国上市，开启了中国使用补体抑制剂治疗PNH溶血的新时代。研究血细胞表面补体活性的检测方法对于未来监测应用补体抑制剂后细胞表面补体活性、预测补体抑制剂疗效非常重要。既往研究主要关注C5b-9在红细胞及白细胞表面的沉积，对其他上游补体片段沉积的研究较少，针对补体片段沉积水平与临床表现相关性的研究则更为罕见。

本研究纳入就诊于我中心的73例PNH患者，其中经典型PNH与AA-PNH综合征各占一半，均有不同程度的贫血及活跃溶血（中位HGB 76 g/L，中位LDH 1 875 U/L），PNH克隆比例较高（中位PNH白细胞克隆比例为94％，中位PNH红细胞克隆比例为46.7％）。通过双抗体染色FCM检测不同血细胞表面补体分子沉积水平，发现患者红细胞、白细胞及血小板表面C5b-9、C3、C4b和FB的沉积均明显高于NC。尽管早期应用ELISA检测的研究表明，C5b-9仅在破碎红细胞和粒细胞膜上检出，完整红细胞表面未结合C5b-9[7],[15]，但此后采用FCM检测的研究显示，溶血患者完整红细胞也结合C5b-9（平均C5b-9^+^红细胞2.2％），并较NC（平均C5b-9^+^红细胞0.9％）明显升高。本研究中患者C5b-9^+^红细胞高于NC，与既往研究一致。另外，既往文献显示，PNH患者补体抑制剂治疗前C3^+^红细胞克隆为阴性，治疗后出现不同水平的升高（中位C3^+^PNH红细胞22.6％）[16]。本研究发现未经补体抑制剂治疗的患者C3^+^红细胞比例较低（平均0.9％），但仍较正常人明显升高（平均0.1％），此结果需未来进一步研究证实。但除了红细胞之外，PNH患者与正常人其他血细胞表面C5b-9及C3沉积、各血细胞表面C4和FB沉积的研究仍缺乏，本研究为未来相关研究提供了一定的依据。

本研究中PNH患者红细胞、白细胞及血小板表面C5b-9的沉积水平都显著高于C3、C4b和FB，提示未经补体抑制剂治疗患者的终末通路补体活性较上游的经典和旁路途径更高。未经补体抑制剂治疗的患者通常IVH显著，但极少出现EVH，因此终末补体通路活性更高[17]。另外，早期应用纯化膜表面结合的C5b-9体外实验发现，C5b-9可抑制经典途径和旁路途径C3转化酶（分别为C4b2a、C3bBb）的形成，并可下调C5转化酶的功能[18]。提示未使用补体抑制剂、终末途径激活、大量C5b-9与细胞膜结合的条件下，上游经典途径（C4b）和旁路途径（C3b、Bb）的活性反馈性下调可能是造成C5b-9的沉积水平高于C3、C4b和FB的原因之一。

进一步分析发现，患者所有补体片段在红细胞表面的沉积水平均明显低于白细胞和血小板。早期文献指出，白细胞和血小板具有多种机制消除其膜表面结合的C5b-9，从而抵御MAC介导的裂解，这些机制包括胞吞或胞吐含C5b-9的囊泡，其中胞吐占主要作用，65％的白细胞膜表面的MAC都能通过胞吐移除[19]。相比之下，红细胞更易发生补体介导的裂解，但有研究表明，红细胞同样能胞吐含C5b-9的囊泡[20]。另有研究指出，由于在一些活化的白细胞亚群中，细胞表面补体调节蛋白（CD46、CD55和CD59）的表达上调，可导致白细胞对补体介导的溶血敏感性较低[21]。2021年，Gurnari等[22]对57例未接受Eculizumab治疗的PNH患者血细胞PNH克隆进行检测，发现血小板对补体介导的溶血的敏感程度低于红细胞，高于白细胞。本研究中，PNH患者红细胞表面补体片段的沉积低于白细胞及血小板，可能是由于红细胞更易发生补体介导的裂解，导致与补体分子结合的红细胞裂解程度较白细胞及血小板更高，使测得的红细胞与补体分子结合的比例更低。

关于补体片段沉积与临床表现的关系，本研究发现，C5b-9^+^红细胞与Ret明显呈正相关，但未见其与LDH的显著相关性，也未发现其余补体片段沉积与临床表现的显著相关性，尽管如此，此结果也提示C5b-9^+^红细胞的活性可能与活跃溶血及造血有关，与临床相符。2009年Risitano等[16]研究了C3^+^PNH红细胞水平与溶血活性（包括LDH及Ret）的相关性，发现在补体抑制剂治疗后患者中，此上游补体分子的沉积水平与Ret水平呈正相关，但与LDH无明显相关性。尚未见未经补体抑制剂治疗的PNH患者溶血活性相关生物学指标（如Ret及LDH）与C5b-9^+^红细胞水平相关性的研究，因此，本研究为后续临床实践中检测此标志物从而监测患者溶血状态提供了新思路。此外，不同PNH亚型的补体沉积水平也不相同，经典型PNH患者C5b-9^+^白细胞、C3^+^白细胞及C3^+^血小板比例（平均值分别为5.5％、3.4％、3.4％）高于AA-PNH综合征（平均值分别为3.9％、1.5％、1.3％）。说明经典型PNH的补体激活水平可能更高，此差异未在红细胞中显示，可能是由于红细胞与补体分子结合后更易溶解，使其表面补体片段阳性率低。

TEE与补体激活的相关性也是近年研究的热点[9]–[10]。2015年，国内一项研究纳入25例PNH患者，PNH患者CD59^-^血小板膜上C5b-9的沉积水平（平均值为17.53％）较PNH患者CD59^+^血小板（平均值为11.33％）及NC的血小板（平均值为10.88％）明显升高[12]–[13]。该研究中PNH患者及NC的中位CD59^-^血小板比例分别为50.58％（23.29％～81.60％）及23.57％（15.58％～29.02％），然而理论上正常人群中不应检出CD59^-^血小板，提示FCM检测膜表面分子比例较实际值偏高，同理推测该研究中测得的C5b-9沉积水平也较实际值偏高。本研究结果中PNH患者及NC的C5b-9^+^血小板比例（平均值分别为4.3％、0.4％）应与实际情况更为接近，但C5b-9在三类血细胞表面的沉积并未显示与TEE的发生情况显著相关。2021年，一项检测PNH患者各血细胞亚群PNH克隆的研究显示，发生TEE者（5例）中位CD59^-^血小板比例显著低于未发生TEE者（18例）（31.05％对75.7％）[22]，也提示血小板与补体片段结合的水平越低，发生TEE的风险可能越高，但该研究例数较少。本研究纳入的患者中18例（24.3％）曾发生过TEE，未发现是否发生TEE患者红细胞上补体沉积水平的差异，可能是其易受补体攻击裂解而与补体分子结合水平较低导致，但TEE组C3^+^白细胞、C4b^+^白细胞及C3^+^血小板水平更低，提示上游通路补体分子（尤其是C3）与血细胞（尤其是白细胞与血小板）结合水平更高者发生TEE的风险可能较低。目前未见C3、C4b、FB沉积水平与TEE风险相关性的研究，还需进一步研究探索。

综上，本研究显示C5b-9、C3、C4b、FB片段在PNH血细胞表面的沉积水平高于正常人，同时对不同补体片段在红细胞、白细胞及血小板上沉积的水平与患者临床症状（特别是发生血栓的风险）及生物学指标的相关性进行了探索。本研究结果为预测患者临床症状提供了参考依据，并为未来应用补体抑制剂后的病情监测及疗效评估提供了新方法。
